# Growing Evidence Linking Microvascular Dysfunction With Heart Failure With Preserved Ejection Fraction

**DOI:** 10.1161/JAHA.116.003259

**Published:** 2016-02-23

**Authors:** Gregory Giamouzis, Erik B. Schelbert, Javed Butler

**Affiliations:** ^1^ Cardiology DivisionUniversity of ThessalyLarissaGreece; ^2^ UPMC Heart and Vascular InstituteUniversity of PittsburghPA; ^3^ Cardiology DivisionStony Brook UniversityStony BrookNY

**Keywords:** Editorials, endothelial dysfunction, heart failure, hypertrophy, nitric oxide, Heart Failure, Magnetic Resonance Imaging (MRI)

Incidence and prevalence of heart failure (HF) with preserved ejection fraction (HFpEF) is rising, with half of all HF patients having a preserved left ventricular ejection fraction (LVEF). These patients have similar mortality rates as patients with HF and reduced EF (HFrEF). HFpEF is observed predominantly in the elderly with a high burden of comorbidities that may contribute to the disease process itself as well as to noncardiac morbidity and mortality.^1^ HFpEF patients have worse outcomes compared to comorbidity‐matched controls.^2^ When compared to healthy and hypertensive controls, patients with HFpEF have greater concentric hypertrophy, atrial enlargement, and diastolic and vascular stiffness that are out of proportion to comorbidities, implying that comorbid conditions alone do not account for the pathophysiology of HFpEF.^3^ Although hypertensive patients without HF and patients with HFpEF share the same increases in arterial and end‐systolic stiffness, the higher degrees of impaired relaxation, diastolic stiffness, and chamber‐ and myocardial‐level contractility in HFpEF, compared to hypertensive and normal controls, underscores worse ventricular mechanics and prognosis.^4^, ^5^

A unifying, but untested, theory of the pathophysiology of HFpEF suggests that comorbidities lead to a systemic inflammation, which triggers endothelial and microvascular dysfunction. This results in diastolic stiffness as well as concentric LV remodeling and fibrosis. Presence of diastolic and vascular dysfunction out of proportion to comorbidities and the lack of neurohormonal antagonism benefitting HFpEF patients might suggest a different pathophysiology of HFpEF compared to those with HFrEF. Alternatively, greater etiological heterogeneity in the less‐specific definition of HFpEF or LVEF‐fibrosis interactions might explain the lack of apparent benefit to neurohormonal antagonism in HFpEF.^6^ Although endothelial dysfunction exists in both HF phenotypes, it is postulated to play a dominant role in the pathophysiology and outcomes of HFpEF. Pathological analyses of the myocardium in HFpEF shows 2 patterns that are thought to contribute to its pathophysiology (Figure). The nitric oxide (NO)‐dependent endothelial dysfunction causes increased myocardial tension attributed to decreased NO bioavailability, and the NO‐independent process promotes collagen production and cross‐linking. Cardiomyocytes of patients with HFpEF have similar force generation, but higher resting tension than normal controls or patients with HFrEF.^7^ This was found to be attributed to hypophosphorylation of titin, which is reversed by increased protein kinase G (PKG) activity.^8^ Titin acts as a spring responsible for myocyte diastolic recoil in early diastole and distensibility in late diastole. NO enhances activity of guanylate cyclase in the conversion of guanylyl triphosphate to cyclic guanylyl monophosphate, which activates protein kinases such as PKG that, in turn, phosphorylate titin, enhancing the diastolic recoil and distensibility during diastole. Thus, oxidative stress and reduced NO bioavailability increases diastolic stiffness through downstream effects on titin.^9^

**Figure 1:**
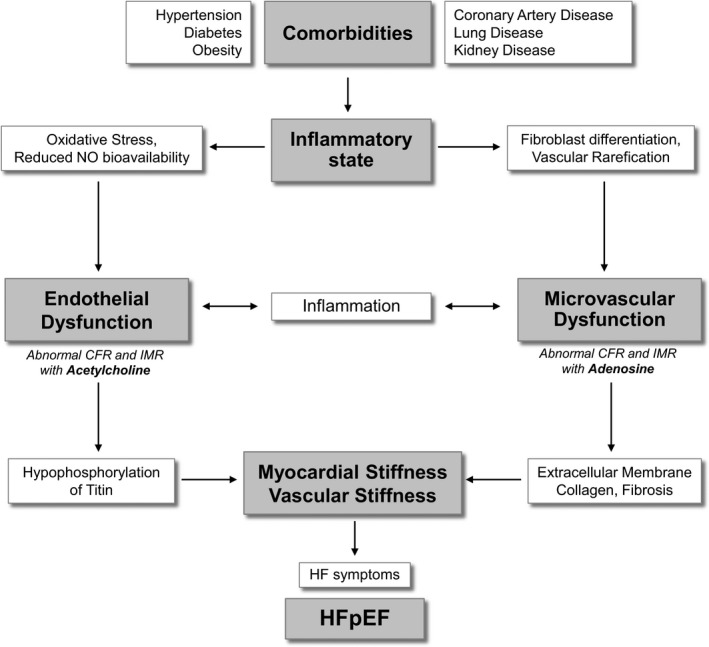
Proposed role of endothelial and microvascular dysfunction in HFpEF. CFR indicates coronary flow reserve; HF, heart failure; HFpEF indicates heart failure with preserved ejection fraction; IMR, index of microcirculatory resistance; NO nitric oxide.

Analysis of hypertensive patients with and without HFpEF reveals elevated serum markers of collagen production and turnover.^10^ HFpEF patients demonstrate more collagen type I and collagen cross‐linking that correlate with higher degrees of diastolic dysfunction.^11^ Stimulation of cardiac fibroblasts in HFpEF patients with transforming growth factor beta results in differentiation into myofibroblasts, which increases type I collagen and reduces matrix metalloproteinase 1, therefore increasing diastolic stiffness through collagen deposition in the extracellular membrane induced by inflammation.^12^ A potential common pathway affecting both stiffness and collagen deposition is that comorbidities contribute to an inflammatory state, resulting in both endothelial dysfunction—resulting in diastolic stiffness—and increased collagen synthesis, leading to fibrosis.

NO‐dependent microvascular dysfunction may be measured using coronary flow reserve (CFR) and possibly index of microcirculatory resistance (IMR) after administration of acetylcholine, whereas NO‐independent microvascular dysfunction may be measured using CFR and IMR after administration of adenosine. Assessment of coronary vascular endothelial function lacks consistency, whereas coronary microvascular function has been only partly assessed in HFpEF. There is therefore an unmet need to determine the presence and severity of endothelial and microvascular dysfunction in patients at risk for, and those with, overt HFpEF. In contrast, vascular endothelial function in peripheral arteries and aortic stiffness has received more attention in HFpEF. Aortic stiffening is greater in HFpEF and vascular endothelial function has been found to be impaired in the digital microvasculature and preserved in large conduit vessels in HFpEF.^13^, ^14^, ^15^ Patients with HFpEF have a depressed endothelial function in the forearm vasculature and microvasculature.^16^

A recent study defined structural changes in full‐thickness myocardial autopsy specimens from 124 subjects with HFpEF and 104 controls dying of noncardiac causes.^17^ Subjects with HFpEF had more cardiac hypertrophy, epicardial coronary artery atherosclerosis, coronary microvascular rarefaction, and myocardial fibrosis and lower microvascular density than controls. Differences in microvascular density and myocardial fibrosis were independent of the severity of epicardial coronary stenosis. Interestingly, myocardial fibrosis was inversely associated with microvessel density. These data support a role of coronary microvascular endothelial inflammation and microvascular rarefaction in the pathophysiology of HFpEF. Both microvascular rarefaction and myocardial fibrosis—including perivascular fibrosis, which limits coronary flow reserve^18^—may uniquely contribute to LV diastolic dysfunction and cardiac reserve function impairment in HFpEF.

In this respect, the study by Kato et al. sheds further light to this important concept. The investigators used phase‐contrast cine‐magnetic resonance imaging (cMRI) of the coronary sinus to assess blood flow at rest and during adenosine triphosphate (ATP) infusion for noninvasive evaluation of CFR in patients with HFpEF, those with hypertensive left ventricular hypertrophy (LVH), and controls.^19^ CFR was calculated as coronary sinus blood flow during ATP infusion divided by coronary sinus blood flow at rest. Impairment of CFR was defined as CFR <2.5. CFR was significantly decreased in HFpEF patients in comparison to hypertensive LVH patients and controls, and independently correlated with natriuretic peptide level. These results indicate that impairment of CFR might be a pathophysiological factor for both development of HFpEF and progression of disease severity.

Endothelial and microvascular dysfunction undoubtedly play an important role in the diastolic abnormalities in HFpEF. There are several questions that remain unanswered, however, the most important being: (1) How important are myocardial endothelial and microvascular dysfunction in the pathogenesis of HFpEF? and (2) What role does myocardial fibrosis play? Are NO‐dependent endothelial dysfunction and inflammation‐induced fibrosis part of the same continuum of disease in HFpEF or separate entities that independently contribute to abnormalities in diastolic function? What are their prognostic implications alone and in combination in patients with HFpEF? Does comorbidity burden increase the likelihood of abnormal CFR in the absence of HFpEF? Is the severity of this abnormal CFR per certain comorbidity burden amplified by the presence of HFpEF? What are the physiological and clinical consequences of myocardial endothelial and microvascular dysfunction in subjects with HFpEF? Do HFpEF patients with NO‐independent microvascular dysfunction have higher levels of biomarkers for fibrosis and a larger amount of diffuse myocardial fibrosis on extracellular volume fraction and T_1_ mapping by cMRI compared to patients with NO‐dependent endothelial dysfunction? And, most important, does this HFpEF subpopulation experience worse outcome compared to the HFpEF subpopulation with NO‐dependent endothelial dysfunction?

There is an unmet need to classify HFpEF patients based on their myocardial vasodilator response as well as characterize their ventricular mechanics, inflammatory and neurohormonal milieu, myocardial substrate, and overall outcomes. To date, no study has evaluated and phenotyped the myocardial substrate in patients with HFpEF and myocardial microvascular dysfunction. Accurate phenotyping may lead to better trial design for future therapeutic advances for treatment of HFpEF. Endothelial and microvascular dysfunction may be early steps in the pathogenesis of HFpEF, and identification of patients with either endothelial or microvascular dysfunction may help identify those without HFpEF at risk for progression to overt HF and other clinical events. Identification and classification of HFpEF based on the presence of endothelial or microvascular dysfunction may identify high‐risk subgroups that may benefit from therapy targeted to the endothelium and/or microvasculature. In this respect, Kato et al. should be congratulated for their important findings that certainly will move this field forward.

## Disclosures

None.
